# Long‐term outcome of supraciliary gold micro shunt in refractory glaucoma

**DOI:** 10.1111/aos.14989

**Published:** 2021-07-27

**Authors:** Michele Figus, Pasquale Loiudice, Andrea Passani, Laura Perciballi, Luca Agnifili, Marco Nardi, Chiara Posarelli

**Affiliations:** ^1^ Ophthalmology Unit Department of Surgical, Medical and Molecular Pathology and Critical Care Medicine University of Pisa Pisa Italy; ^2^ Ophthalmology Clinic Department of Medicine and Aging Science University G. D’Annunzio of Chieti‐Pescara Chieti Italy

**Keywords:** gold micro shunt, long‐term follow‐up, refractory glaucoma, supraciliary shunt

## Abstract

**Purpose:**

To evaluate the 10‐year follow‐up efficacy and safety of gold micro shunt (GMS) in patients with refractory glaucoma, and the potential risk factors for failure.

**Methods:**

Retrospective data analyses based on medical records from 55 patients who underwent GMS implant for refractory glaucoma between March 2007 and April 2008. The primary outcome measure was the cumulative probability of success defined as intraocular pressure (IOP) below 21 mmHg together with a 33% lowering of the baseline IOP with (qualified) or without (complete) topical medications, no reoperation for glaucoma or loss of light perception.

**Results:**

Mean IOP 10 years after the GMS implantation was 21.6 ± 5.1 mmHg with 2.7 ± 0.7 drugs. Qualified success was achieved in 8/55 patients (14.5%) with a mean of 2.9 ± 0.8 drugs at 5 years and in 2/55 patients (3.6%) with a mean of 2.7 ± 1.0 drugs at 10 years. None of the patients reached complete success at five years from surgery. The cumulative probability of complete success was 14%, 9% and 0% at 1, 2 and 5 years, respectively, and 72%, 67%, 36% and 3.6% at 1, 2, 5 and 10 years, respectively, for qualified success criterion. Baseline IOP for complete success, number of baseline medication for qualified success and age at the time of GMS implantation for both criteria were risk factors significantly associated with failure.

**Conclusion:**

A very low long‐term survival rate of GMS in refractory glaucoma was found. Most patients did not reach the IOP success criteria of the study, even with the re‐introduction of medications, leading to the need for further surgical procedures.

## Introduction

The management of refractory glaucoma still represents a challenge for ophthalmologists. Mostly, refractory glaucoma patients have a history of failed standard filtration surgery or drainage implant procedures and show a progression of visual field damage despite maximal tolerated medical therapy. Furthermore, long‐term medical therapy to control intraocular pressure (IOP) induces alteration of ocular surface, which decreases the likelihood of further filtration surgery survival (Broadway et al. 1998). For these reasons, an alternative approach that did not implicate the formation of a filtering bleb was sought. Supraciliary space accounts for 10–15% of physiological aqueous humour outflow and was identified as a possible alternative drainage strategy (Nesterov et al. 1979; Townsend & Brubaker 1980). This pathway was also targeted by drugs such as atropine and prostaglandin analogues, as confirmed by human and animal models (Bill 1969; Ziai et al. 1993).

Several draining devices such as iStent Supra (Glaukos Corporation, Laguna Hills, CA, USA), CyPass Micro‐Stent (Transcend Medical Inc., Menlo Park, CA, USA), Aquashunt (OPKO Health Inc., Miami, FL, USA) and STARflo Glaucoma Implant (iSTAR Medical SA, Wavre, Belgium) (Hoeh et al. 2013; Oatts et al. 2013; Cseke et al. 2016; Gigon & Shaarawy 2016; Hoeh et al. 2016; Figus et al. 2017; Grisanti et al. 2018; Myers et al. 2018) have been developed in the past two decades to connect the anterior chamber to the supraciliary space, exploiting the natural pressure gradient that exists between these two compartments (Emi et al. 1989).

The gold micro shunt (GMS, SOLX Ltd, Boston, MA, USA) is a non‐valved flat‐plate drainage device made of 99.95% 24‐karat medical grade gold and was one of the first supraciliary devices commercialized. It provides an alternative pathway for aqueous drainage avoiding bleb formation and, therefore, the most common filtration surgery complications such as bleb leakage and bleb‐related endophthalmitis, hypotony, choroidal effusion and subconjunctival fibrosis (Rulli et al. 2013; Chen et al. 2014).

In a pilot study, Melamed and associates reported that the GMS implant was safe and well‐tolerated, with satisfactory IOP control at a mean follow‐up of 11.7 months (Melamed et al. 2009). Hueber et al. retrospectively analysed the reports of 31 patients diagnosed with severe glaucoma, up to 4 years after the GMS implant concluding that it was not an effective method to lower IOP (Hueber et al. 2013). Skaat and co‐workers conducted a randomized prospective clinical trial comparing 5‐year follow‐up outcomes of GMS with Ahmed glaucoma valve in refractory glaucoma, reporting similar success rates between surgeries (Skaat et al. 2016). More recently, Tanito and Chihara observed a 23% of IOP reduction at 1 year after the GMS implantation in an open‐angle glaucoma Japanese population (Tanito & Chihara 2017).

In this study, we aimed to evaluate the 10‐year follow‐up efficacy and safety of GMS in patients with refractory glaucoma, and the potential risk factors for failure.

## Materials and Methods

This monocentric study was performed in adherence to the tenets of the Declaration of Helsinki; all patients signed an informed consent form before enrolment. We received approval from the local Institutional Review Board (CEAVNO, Comitato Etico Area Vasta NordOvest, Tuscany, Italy: Parere_17680_Figus).

The device considered in this study was the first version of GMS. It was a thin gold plate 3.2 mm wide, 5.2 mm long and 44 µm thick containing 19 channels (nine open and 10 closed) with a lumen width of 24 µm and a height of 50 µm (Melamed et al. 2009). We enrolled 55 eyes of 55 patients affected by refractory glaucoma, defined as uncontrolled IOP (>21 mmHg) despite maximal antiglaucoma medical therapy, previously failed surgical treatment, or a combination of both; all GMS implantations were conducted in the University Hospital of Pisa, Italy, from March 2007 to April 2008. All the surgeries were performed by the same expert surgeon (M.N.) who was adequately trained before the study began. All patients received the same version of the GMS.

Inclusion and exclusion criteria have been described in our previous study in which the 55 consecutive cases were prospectively evaluated up to 2‐year follow‐up (Figus et al. 2011). Briefly, angle‐closure glaucoma, neovascular glaucoma, pregnancy, known allergy to medications needed during and after surgery, current use of any other investigational drug or device, combined surgical procedures (i.e. Micro Shunt and cataract surgery) and other ocular comorbidities that could interfere with follow‐up measurements were considered exclusion criteria.

We retrospectively analysed the 5‐ and 10‐year data from those patients to assess the long‐time efficacy and safety of the GMS device. Analysis of our digital database started in January 2020 and ended in May 2020. Best‐corrected visual acuity (BCVA) converted in logarithm of the minimum angle of resolution (logMAR), IOP measured with Goldmann applanation tonometer, number of glaucoma medications, number of visits performed during follow‐up, number of additional surgical procedures and perimetric parameters (mean deviation and pattern standard deviation) were registered up to 10 years after implantation and were included in the statistical analysis.

We used the success criteria as previously defined: (i) complete success was defined as IOP below 21 mmHg together with at least a 33% reduction in the baseline IOP without any topical medications; (ii) qualified success was defined as IOP below 21 mmHg together with a reduction of the 33% of the baseline IOP with or without topical medications (Figus et al. 2011). Treatment failure was defined as IOP ≥21 mmHg or less than 33% reduction with respect to baseline on two consecutive follow‐up visits, IOP <5 mmHg on two consecutive follow‐up visits, reoperation for glaucoma or loss of light perception.

Statistical analysis was performed using SPSS statistical package (version 25 for Windows, IBM, Armonk, USA). Kaplan–Maier survival analysis was applied to assess the long‐term outcomes for each of the two success criteria. Cumulative probability of success was defined as the probability obtained by multiplying probabilities of survival at each time point of follow‐up. Univariate and multivariable Cox proportional‐hazards regression analyses were used to evaluate risk factors for failure. Risk factors for failure were age, gender, eye laterality, baseline IOP and baseline number of medications. All subjects had bilateral glaucoma but only one eye per patient received GMS implant. Risk factors with a p value < 0.2 in univariate analysis were included in the multivariable analysis. A p value of < 0.05 was considered statistically significant.

## Results

Demographics of patients were previously described and was displayed in Table 1 (Figus et al. 2011).

**Table 1:** Baseline characteristics of patients with refractory glaucoma treated with gold micro shunt implant.

Parameter	Value
Patients	55
Eyes	55
Age (years)	
Mean ± SD	64.1 ± 15.9
Range	18–86
Gender	
Male/female	35/20
Ethnicity	Caucasian
Eye laterality	
Right/left	31/24
Preoperative IOP	
Mean ± SD	30.8 ± 8.8
Range	22–58
N. of baseline medications	
Mean ± SD	2.8 ± 1.1
Range	2−4
Best corrected visual acuity (logMAR)	0.047 ± 0.03
Visual field mean deviation (dB)	−10.82 ± 8.41
N. of previous glaucoma surgery	
Mean ± SD	1.9 ± 0.7
Range	1–5
Lens status (%)	
Phakic	12 (21.8)
Pseudophakic	40 (72.7)
Aphakic	3 (5.5)
Other previous ocular surgery	
Vitrectomy for macular pucker	2
Vitrectomy for retinal detachment	1
Keratoplasty	2

IOP = intraocular pressure; N = number; SD = standard deviation.

Among the 55 patients originally implanted in our site, 15 subjects did not reach the 2‐year follow‐up visit that we reported previously (Figus et al. 2011), 18 patients attended the 10‐year follow‐up visit but six patients had died at this time of the survey. Overall, data of 40 patients (26 men and 14 women) were available and analysed for study purposes. The mean age of the population 10 years after surgery was 73.2 ± 12.01 (range 42–89) years, and the mean BCVA was 1.30 ± 1.06 LogMAR (range 0.3–3 logMAR).

Baseline BCVA was 0.047 ± 0.03 LogMAR, and preoperative visual field mean deviation was −10.82 ± 8.41 dB. Because of the advanced stage of the disease of our population, perimetric data at 5 years and 10 years postoperatively were not available. Mean IOP 10 years after the GMS implantation was 21.6 ± 5.1 mmHg with 2.7 ± 0.7 drugs (Table 2).

**Table 2:** Intraocular pressure in patients with refractory glaucoma treated with gold micro shunt implant according to qualified and complete success criteria.

Group	Data	Baseline	1 day	1 week	1 month	3 months	6 months	1 year	2 years	5 years	10 years
Total	Patients at each follow‐up visit, *n* (%)	55 (100)	55 (100)	55 (100)	55 (100)	51 (92.7)	47 (85.5)	42 (76.4)	40 (72.7)	34 (61.8)	18 (32.7)
	IOP (mean ± SD)	30.8 ± 8.8	12.6 ± 5.85	17.1 ± 7.87	16.2 ± 7.22	16.2 ± 6.23	14.8 ± 6.35	15.5 ± 3.73	13.7 ± 2.98	19.9 ± 3.9	21.6 ± 5.1
	IOP reduction, *n* (%)		18.2 (59.1)	13.7 (41.9)	13.9 (45.1)	14.6 (47.4)	16.0 (51.9)	15.3 (49.7)	13.9 (50.4)	10.9 (28.1)	9.2 (29.9)
	Glaucoma medications (mean ± SD)	2.8 ± 1.1	0.0 ± 0.0	0.6 ± 0.3	0.9 ± 0.4	1.1 ± 0.4	1.3 ± 0.6	1.4 ± 0.8	1.5 ± 0.8	2.1 ± 0.8	2.7 ± 0.7
Qualified success (10 years)	Eyes, *n* (%)	2 (3.6)									
	IOP (mean ± SD)	27.6 ± 7.15	11.5 ± 5.59	16.5 ± 7.63	15.1 ± 4.71	15.8 ± 2.97	14.1 ± 4.55	15.0 ± 3.45	13.7 ± 2.98	17.9 ± 2.4	16.18 ± 2.5
	IOP reduction, *n* (%)		16.1 (58.3)	11.1 (40.2)	12.5 (45.3)	11.8 (42.8)	13.5 (48.9)	12.6 (45.7)	13.9 (50.4)	9.7 (35.1)	11.4 (41.3)
Success		55	55	55	51	47	42	40	37	8	2
Censored	Lost to follow‐up	0	0	0	0	4	4	5	2	4	12
	Dead	0	0	0	0	0	0	0	0	2	4
Failure	IOP ≥ 21 mmHg or <33%	0	0	0	2	4	1	0	0	9	5
	Reop. for glaucoma	0	0	0	0	0	4	2	3	14	8
	IOP <5 mmHg	0	0	0	2	0	0	0	0	0	0
	Loss of light perception	0	0	0	0	0	0	0	0	3	3
Complete success (10 years)	Eyes, *n* (%)	0									
	IOP (mean±SD)	26 ± 3.46	10 ± 4.00	14 ± 4.00	10 ± 4.00	14.4 ± 3.21	14 ± 3.46	18.3 ± 3.58	16.4 ± 2.91	–	–
	IOP reduction, *n* (%)		16.0 (61.5)	12.0 (46.2)	16.0 (61.5)	11.6 (44.6)	12.0 (46.2)	7.7 (29.6)	9.6 (36.9)		
Success		55	55	55	41	39	22	8	3	0	0
Censored	Lost to follow‐up	0	0	0	0	4	4	5	2	4	12
	Dead	0	0	0	0	0	0	0	0	2	4
Failure	IOP ≥ 21 mmHg or <33%	0	0	0	12	12	21	32	34	17	9
	Reop. for glaucoma	0	0	0	0	0	4	2	3	14	8
	IOP <5 mmHg	0	0	0	2	0	0	0	0	0	0
	Loss of light perception	0	0	0	0	0	0	0	0	3	3

Complete success was defined as IOP below 21 mmHg together with a reduction of at least the 33% of the baseline IOP without any topical medications; qualified success was defined as IOP below 21 mmHg together with a reduction of at least the 33% of the baseline IOP with or without topical medications. Failure was defined as IOP≥21 mmHg or <33% reduction with respect to baseline on two consecutive follow‐up visits, IOP < 5 mmHg on two consecutive follow‐up visits, reoperation for glaucoma or loss of light perception.

*n* = number; IOP = intraocular pressure; SD = standard deviation.

The difference in IOP compared with baseline was statistically significant both at 5‐year (p < 0.001, Wilcoxon signed‐rank test) and at 10‐year (p < 0.001) follow‐up. The number of antiglaucoma medications was not significantly different neither at 5 years (p = 0.063, Wilcoxon signed‐rank test) nor at 10 years (p = 0.132) postoperatively.

Nine out of 55 patients underwent reoperation for glaucoma during the first 2 years from the implant. Later, the other 22 subjects required additional glaucoma surgeries, with a mean of 1.86 procedure per patient (trabeculectomy or Ahmed valve implant). Ten patients underwent shunt removal, and one patient underwent shunt repositioning. The incidence of shunt removal was noted to be the highest during the first 5 years from GMS implantation; four shunts were surgically removed in 2010 (2–3 years from surgery), three shunts during 2011 (3–4 years from surgery) and three GMSs during 2012 (4–5 years from surgery). The main cause of GMS removal was the superficialization of the shunt (6 cases) with consequent conjunctival erosion and corneal endothelium alterations. In the other four cases (three cases during 2011 and one during 2012), at the same time of the additional glaucoma surgery, the surgeon intraoperatively inspected the GMSs. As he observed that the GMSs were completely encapsulated by a fibrotic membrane, he decided to proceed with the removal of the shunts. In one case, we also recorded a spontaneous expulsion of the GMS. In this unusual instance, the shunt was spontaneously expelled by the conjunctiva and the patient came for a visit bringing back the shunt.

**Fig. 1:**
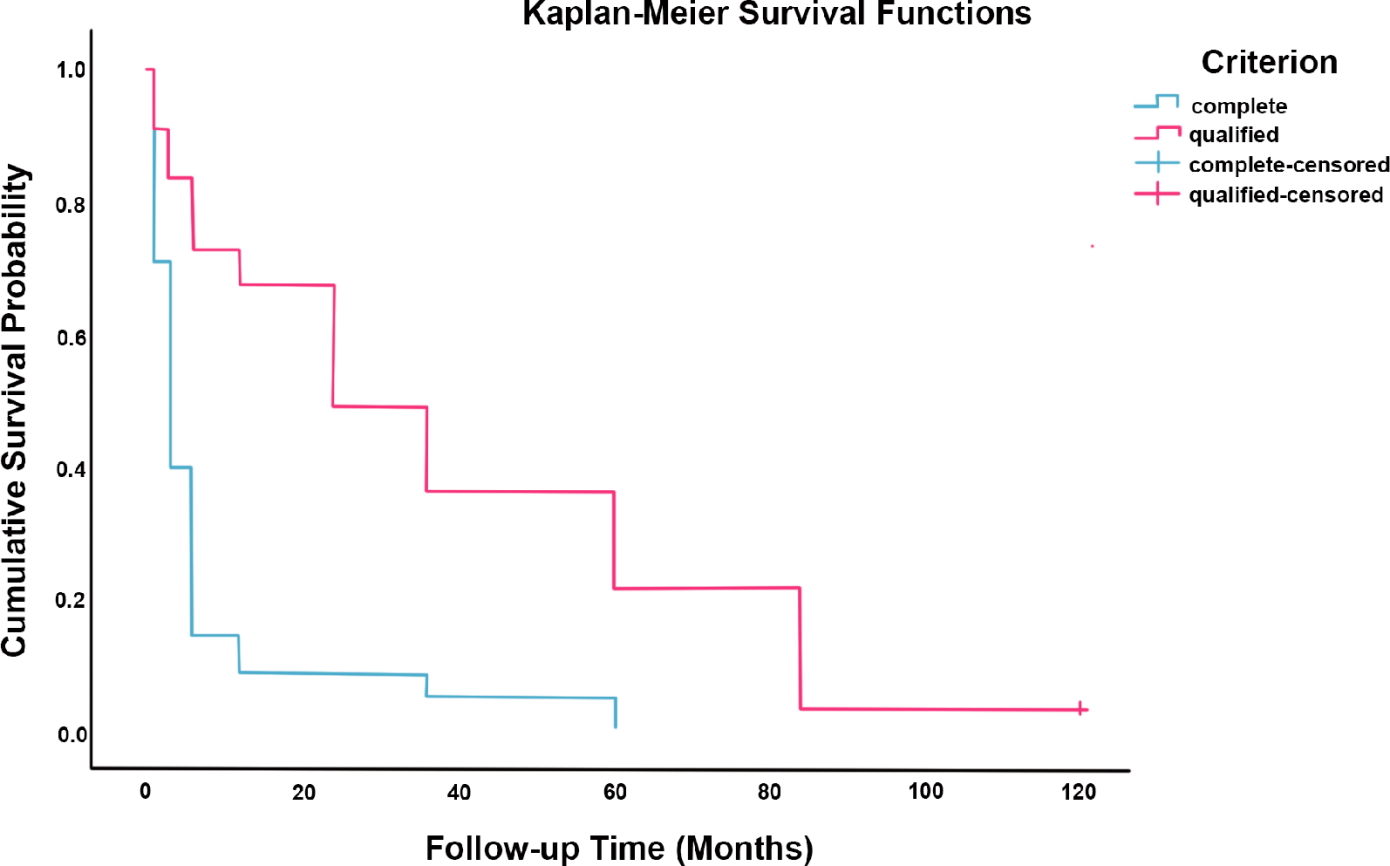
Kaplan–Meier survival estimates for criterion. Kaplan–Meier survival curve showing the survival probability of gold micro shunt in refractory glaucoma. Complete success: IOP below 21 mmHg together with a reduction of at least 33% of the baseline IOP without any topical medications; qualified success: IOP below 21 mmHg together with a reduction of at least 33% of the baseline IOP with or without topical medications. IOP = intraocular pressure.

The cumulative probability of success was 14%, 9% and 0% at 1, 2 and 5 years, respectively, for the complete success criterion 72%, 67%, 36% and 3.6% at 1, 2, 5 and, 10 years, respectively, for qualified success criterion (Fig. 1).

In our population, qualified success at five years was achieved in 8/55 patients (14.5%) with a mean of 2.9 ± 0.8 drugs, whereas at 10 years was achieved in only 2/55 patients (3.6%) with a mean of 2.7 ± 1.0 drugs. None of our patients reached complete success at five years from surgery.

Reasons for failure in the period between 2 years and 5 years after the GMS implant, for complete success, were loss of light perception in six cases, need for further surgery in 22 cases; the remaining subjects did not reach a lowering in IOP of at least 33% from baseline without medications.

For both success criteria, potential risk factors identified with univariate analyses (p < 0.2) were as follows: age, gender, number of medications at baseline and baseline IOP. In the multivariable Cox proportional‐hazards model, the risk factors significantly associated with failure were age at the time of GMS implantation for both criteria, with both age per decade and age as a continuous variable, baseline IOP for complete success and number of baseline medication for qualified success (Table 3). Older age at the time of the implant, higher value of baseline IOP and higher number of baseline medications are associated with increased risk of failure.

**Table 3:** Risk factor for failure; results from multivariable Cox regression analysis in patients with refractory glaucoma treated with gold micro shunt implant.

Risk factor	Complete success			Qualified success		
	HR	95% CI	p	HR	95% CI	p
Age (per decade)	0.393	0.277–0.558	**0.006**	0.269	0.174–416	**0.027**
As continuous variable	0.939	0.921–0.958	**0.048**	0.902	0.873–0.932	**0.016**
Baseline IOP	0.869	0.777–0.971	**0.013**	0.997	0.962–1.033	0.872
Baseline *n*. of medications	0.812	0.343–1.019	0.636	0.197	0.082–0.474	**0.001**

Complete success was defined as IOP below 21 mmHg together with a reduction of at least the 33% of the baseline IOP without any topical medications; qualified success was defined as IOP below 21 mmHg together with a reduction of at least the 33% of the baseline IOP with or without topical medications. Significant p values are in bold.

CI = confidence interval; HR = hazard ratio; IOP = intraocular pressure; *n* = number.

In terms of safety, 6/55 shunts (10.9%) were surgically removed due to the superficialization, whereas 1/55 shunt (1.8%) was surgically repositioned. In 1/55 case (1.8%), we recorded a spontaneous expulsion of the shunt from the conjunctiva without any need for surgical assistance. We registered 1/55 case (1.8%) of endophthalmitis and 6/55 cases (10.9%) of corneal endothelial decompensation. Encapsulation of the shunt occurred in 4/55 cases (7.2%) and led to surgical shunt removal with additional glaucoma surgery. In our series, we also recorded 2/55 cases (3.6%) of recurrent uveitis that led to Ahmed valve implantation to control IOP. No other long‐term adverse events were registered.

## Discussion

We retrospectively investigated the safety and efficacy of GMS in patients with refractory glaucoma up to 10 years from the implantation. Gold micro shunt (GMS) was one of the first glaucoma devices produced to exploit the supraciliary space to lower IOP. The supraciliary pathway was considered as a promising alternative for aqueous drainage to obtain an efficient and safe bleb‐less surgery. Low inflammatory response, negative hydrostatic pressure and easy access through the angle were the main benefits of the supraciliary path that has been targeted by many other glaucoma devices (Oatts et al. 2013; Gigon & Shaarawy 2016; Hoeh et al. 2016; Grisanti et al. 2018; Myers et al. 2018).

Several hypotheses have been formulated regarding the mechanism of the action of GMS. As supposed by Melamed et al. (2009), once the aqueous humour reaches the supraciliary space, it drains via choroidal vessels and through the sclera. The latter drainage route was also demonstrated by using anterior segment optical coherence tomography and in vivo confocal microscopy, which documented in functioning cases a spongy aspect of the sclera along with epithelial microcysts in the conjunctiva overlying the GMS (Mastropasqua et al. 2010).

The initial data from the pilot study of Melamed et al. in 2009 (Melamed et al. 2009) showed a good efficacy of GMS with a mean decrease in IOP of 9 mmHg at 1‐year follow‐up, with or without medications. More recently, in a population of Japanese patients with open‐angle glaucoma, Tanito and Chihara reported a mean IOP of 16.4 ± 5.8 mmHg (23% reduction from baseline) with the need to use 2.1 ± 1.1 medications at 1 year postoperatively (Tanito & Chihara 2017). In our series, we observed a mean reduction of 15.3, 13.9, 10.9 and 9.2 mmHg, at 1, 2‐, 5‐ and 10‐year follow‐up, respectively. In terms of surgical success, Melamed et al. reported a complete success (IOP between 5 and 22 mmHg without medications) in 13.2% of cases, and a qualified success in 79% of cases at a 1‐year follow‐up. Similar results were reported by Mastropasqua et al. (Mastropasqua et al. 2010) (57% qualified success after 15 months) and Figus et al. (2011) (67.3% qualified success after 2 years from the implant). Skaat et al. found a qualified success in 77.8% of cases at a 5‐year follow‐up comparing Ahmed glaucoma valve with 24 µm and 48 µm GMSs. These initial promising results were not further confirmed by Hueber et al. (Hueber et al. 2013). Despite the use of the last version of the device, GMS Plus, in which microchannels have a height of 40 μm and a width of 50 μm, they observed a failure rate of 71% at 1 year and 90% at 2 years after implantation. In our series, we found a cumulative probability of complete success of 14%, 9% and 0% at 1, 2 and 5 years, respectively, and 72%, 67%, 36% and 3.6% at 1, 2, 5 and 10 years, respectively, for qualified success. Although the mean IOP at 5‐ and 10 years after the GMS implant was lower than baseline, this was not enough to fulfil the success criteria considered in this study. Furthermore, six subjects were considered failures as they lost light perception.

Discrepancies in terms of efficacy between studies could be partially explained by differences in patient selection, surgical technique, the definition of success criteria, study design and shunt model. Patients with a history of previous failed surgical procedures, and subjects who received GMS as a primary surgery were all included both in the study of Melamed et al., and in that of Hueber et al. (Melamed et al. 2009; Hueber et al. 2013); conversely, in our series and in the study of Skaat et al., only subjects with refractory glaucoma were enrolled (Skaat et al. 2016). Tanito and Chihara also included patients who underwent GMS implantation combined with cataract surgery (Tanito & Chihara 2017). Surgical success has been alternatively considered as an IOP value between 5 mmHg and 21 mmHg, or a reduction of 20% or 33% of baseline IOP, no need for further procedures or a combination of these.

The very low long‐term survival rate we found in our series could be affected by our glaucoma population of refractory glaucoma and may be due to the fibrotic processes taking place after GMS implantation. Histological findings demonstrated the presence of a thick connective capsule‐like reaction surrounding both the ends of the device and a connective tissue filling the inner channels in non‐functioning cases (Agnifili et al. 2012). Fibrosis may be a consequence of the activation and proliferation of fibroblasts of supraciliary space excited by surgical manipulation and/or migration of Tenon’s fibroblasts throughout the scleral incision (Agnifili et al. 2012). Furthermore, increased levels of inflammatory cytokines, such as transforming growth factor‐beta 2, have been found in higher amounts in the aqueous humour of patients with glaucoma (Tripathi et al. 1994). Previous evidence has documented that the aqueous humour of patients with failed filtering surgery contains higher levels of tumour necrosis factor‐alpha and interleukin‐6, compared with the aqueous humour of patients who underwent successful surgery (Picht et al. 2001; Cvenkel et al. 2010; Pantalon et al. 2019). Modifications induced by the GMS into the anterior chamber, aqueous humour and supraciliary space may contribute to elucidate possible mechanisms of failure. Because of the elevated rates of failure and the serious complications reported in some case postoperatively, the GMS never received Food and Drug Administration approval (Hueber et al. 2013; Pereira et al. 2021).

Risk factors for failure identified in our multivariate analysis were age at the time of surgery, baseline IOP value for complete success and baseline number of drugs for qualified success. It has been postulated that GMS could have a role in cases of refractory glaucoma even if the small sample size of the published studies limited a subgroup analysis.

Supraciliary shunts were designed in the effort to lower the IOP avoiding the bleb‐related complication of traditional procedures. A biocompatibility study in rabbit showed the absence of elicited tissue reaction or encapsulation of the GMS (Orozco et al. 2004). In their pilot study, Melamed et al. reported one case (3%) of shunt exposure, one case of synechia formation, mild hyphaema in six patients (16%), moderate hyphaema in two patients (5%) and one case of exudative inferior retinal detachment. Mostly, the literature published up to now reported minor to moderate grade complications. Overall, the most common complication associated with GMS implant was transient bleb formation. Other complications included hyphaema, shallow anterior chamber, hypotony, anterior chamber cells, fibrin reaction, recurrent uveitis, shunt‐iris synechia, macular oedema, cataract progression, shunt malposition, encapsulation and extrusion. In our long‐term follow‐up, the GMS was removed in 10 eyes (six due to the superficialization, and four to shunt encapsulation). We also observed one case of endophthalmitis, six cases of corneal endothelial decompensation and one case of spontaneous expulsion of the shunt. The latter may be due to an extreme superficialization and retroposition of the shunt that allowed a spontaneous expulsion of the shunt through the conjunctiva without any other damage to ocular structures.

In this study, we observed a very low long‐term survival rate of GMS implant at 10‐year follow‐up. This result should be read in the context of patients with refractory glaucoma and a mean of 1.9 (range 1–5) previous glaucoma surgeries. Furthermore, the subjects enrolled in the present study had an advanced stage of the disease and probably, even if treated differently, they would have had a low chance of long‐term success.

Luzu and colleagues found a cumulative success rate of 45.1% at 5 years after AGV implant in refractory glaucoma subjects (Luzu et al. 2020). Similarly, Souza and co‐workers found a cumulative probability of qualified success of 49% at 5 years after Ahmed glaucoma valve (AGV) implantation in refractory glaucomas (Souza et al. 2007). The above‐mentioned authors identified a prior glaucoma surgery as a risk factor for failure.

Primary glaucoma procedures have a higher probability of success. In open‐angle glaucoma patients, primary deep sclerectomy had a success rate at 7 years of 54% for IOP ≤ 18 mmHg, 29.8% for IOP ≤ 15 mmHg and 10% for IOP ≤ 12 mmHg (Rabiolo et al. 2021). In diabetic patients who underwent primary trabeculectomy for primary open‐angle glaucoma, the cumulative survival rates were 66.7 ± 8.4% at 36 months, 57.8 ± 9.3% at 60 months and 50.6 ± 10.6% 84 months (Law et al. 2013). In a retrospective study of patients with open‐angle, which includes primary open‐angle, pseudoexfoliation and pigmentary dispersion glaucoma who underwent trabeculectomy with mitomycin C, the cumulative probability of success (IOP(13 mm Hg and a reduction of IOP>30% from baseline) at 5 years was 22% (Tran et al. 2009).

In a population of eyes with intractably raised IOP following pars plana vitrectomy, the cumulative probability of qualified success after AGV implant was 80.7% at 5 years and 74% at 10 years (Pandav et al. 2021). A recent study evaluated the effectiveness of Preserflo Microshunt in patients with primary open‐angle glaucoma and found a qualified success of 82.6% at 5 years (18 patients) (Batlle et al. 2021).

The main limitations of this study were its retrospective design and the limited data availability at the final follow‐up. Risk factors such as refractive status, glaucoma diagnosis and visual field disease stage were not included in univariate analysis. In the current study, we reported the surgical results of one surgeon in one centre; there was no control group with another surgical method. Furthermore, we included in our study patients who underwent previous surgery other than glaucoma surgery (vitrectomy, keratoplasty). Although pars plana vitrectomy and penetrating keratoplasty could be considered risk factors for ocular hypertension and glaucoma (Rossi & Ripandelli 2020; González‐Pérez et al. 2021), a cumulative probability of qualified success of 74% at 10 years after AGV implant was reported in a population of eyes with intractably raised IOP following pars plana vitrectomy (Rabiolo et al. 2020). Similarly, a glaucoma drainage device was able to control intractable glaucoma after penetrating keratoplasty in 70% of eyes at 10 years postoperatively (Purtskhvanidze et al. 2021). However, despite limitations, the present study may provide notable additional elements regarding the GMS, as it reported information coming from a long‐term follow‐up, and contributed to better clarify the role of GMS in refractory glaucoma.

In conclusion, we found a very low long‐term survival rate of GMS in cases of refractory glaucoma, and some major complications were observed. Most patients did not reach the IOP success criteria of the study, even after the re‐introduction of antiglaucoma medications, leading to the need for further surgical procedures.
